# Supplementary material for: Fewer Children in Families Associated with Lower Odds of Early Childhood Caries: A Sample from Three Countries
Source: Int J Environ Res Public Health. 2023 Jan 26;20(3):2195. doi: 10.3390/ijerph20032195 (PMC9916343; doi:10.3390/ijerph20032195)
Supplement: Supplementary file 1 [file ijerph-20-02195-s001.zip › ijerph-2165576-supplementary.pdf]

Supplementary Materials

In Table S1, among those with decayed teeth, a one-unit increase in the number of children was found to significantly increase the expected number of child total dmft by a factor of 1.06. Among all children, a one-unit increase in the number of children was found to decrease the odds of being in the zero dmft group by a factor of 0.74.

Table S1. Sensitivity analysis: unadjusted zero-inflated Poisson model.

Oral Health Outcome	Count Model Estimate [a] (95% CI)	Count Model *p*-Value	Inflation Model Estimate [a] (95% CI)	Inflation Model *p*-Value
Number of decayed, missing and filled teeth (dmft)	1.06 (1.03–1.08)	<0.0001	0.74 (0.65–0.82)	<0.0001

[a] Percentile-based confidence interval.

In Table S2, among those with decayed teeth, the expected number of child total dmft for children living in an urban area is 0.95 times that of children living in a rural area, and the expected number for males is 1.09 times that of females. A one-unit increase in age significantly increases the expected number of child total dmft by a factor of 1.09 and significantly decreases the odds of being in the zero dmft group by a factor of 0.56.

Table S2. Sensitivity analysis: adjusted zero-inflated Poisson model by covariates.

Impact of Covariates on dmft	Count Model Estimate [a] (95% CI)	Count Model *p*-Value	Inflation Model Estimate [a] (95% CI)	Inflation Model *p*-Value
Site [b]	0.95 (0.86–1.05)	–	1.16 (0.84–1.63)	0.372
Sex [b]	1.09 (1.00–1.17)	0.0003	1.08 (0.83–1.43)	0.555
Age	1.09 (1.06–1.13)	<0.0001	0.56 (0.50–0.62)	<0.0001

[b] *Site reference group: rural; sex reference group: female. [a] Percentile-based confidence interval.

In Table S3, among those with decayed teeth, a one-unit increase in the number of children was found to significantly increase the expected number of child total dmft by a factor of 1.15 in Vietnam. Among all children, a one-unit increase in the number of children was found to significantly decrease the odds of being in the zero dmft group by a factor of 0.77 in Nepal.

Table S3. Sensitivity analysis: adjusted zero-inflated Poisson model by country.

By Country	Count Model Estimate [b] (95% CI)	Count Model *p*-Value	Inflation Model Estimate [b] (95% CI)	Inflation Model *p*-Value
Ecuador [a]	—	—	—	—
Nepal	1.06 (0.97–1.15)	0.0208	0.78 (0.59–0.96)	0.024
Vietnam	1.11 (1.03–1.19)	<0.0001	0.80 (0.55–1.11)	0.185

[a] Adjusted ZIP model could not converge. [b] Percentile-based confidence interval.
